# Evidence for Health I: Producing evidence for improving health and reducing inequities

**DOI:** 10.1186/s12961-016-0087-2

**Published:** 2016-03-14

**Authors:** Anne Andermann, Tikki Pang, John N Newton, Adrian Davis, Ulysses Panisset

**Affiliations:** Department of Family Medicine and Department of Epidemiology, Biostatistics and Occupational Health, Faculty of Medicine, McGill University, Montreal, Canada; Lee Kuan Yew School of Public Policy, National University of Singapore, Singapore, Singapore; Institute of Population Health, Faculty of Medical and Human Sciences, University of Manchester, Manchester, England; Public Health England, London, England; Department of Preventive and Social Medicine-Health Policy, Faculty of Medicine, Federal University of Minas Gerais, Belo Horizonte, Brazil; Evidence Informed Policy Network (EVIPNet) Steering Group, World Health Organization, Geneva, Switzerland

**Keywords:** Decision-making, Evidence-based medicine, Health equity, Health policy, Public health, Research

## Abstract

In an ideal world, researchers and decision-makers would be involved from the outset in co-producing evidence, with local health needs assessments informing the research agenda and research evidence informing the actions taken to improve health. The first step in improving the health of individuals and populations is therefore gaining a better understanding of what the main health problems are, and of these, which are the most urgent priorities by using both quantitative data to develop a health portrait and qualitative data to better understand why the local population thinks that addressing certain health challenges should be prioritized in their context. Understanding the causes of these health problems often involves analytical research, such as case-control and cohort studies, or qualitative studies to better understand how more complex exposures lead to specific health problems (e.g. by interviewing local teenagers discovering that watching teachers smoke in the school yard, peer pressure, and media influence smoking initiation among youth). Such research helps to develop a logic model to better map out the proximal and distal causes of poor health and to determine potential pathways for intervening and impacting health outcomes. Rarely is there a single ‘cure’ or stand-alone intervention, but rather, a continuum of strategies are needed from diagnosis and treatment of patients already affected, to disease prevention, health promotion and addressing the upstream social determinants of health. Research for developing and testing more upstream interventions must often go beyond randomized controlled trials, which are expensive, less amenable to more complex interventions, and can be associated with certain ethical challenges. Indeed, a much neglected area of the research cycle is implementation and evaluation research, which often involves quasi-experimental research study designs as well as qualitative research, to better understand how to derive the greatest benefit from existing interventions and ways of maximizing health improvements in specific local contexts. There is therefore a need to alter current incentive structures within the research enterprise to place greater emphasis on implementation and evaluation research conducted in collaboration with knowledge users who are in a position to use the findings in practice to improve health.

“*Even if the cure for HIV was one glass of clean water, we wouldn’t be able to cure the world.*” – Technical Officer at the World Health Organization, Geneva, Switzerland

## Background

To help people make better-informed decisions about improving health and reducing health inequities, an important question is, what evidence is needed in supporting these decisions [1]? There is a large body of biomedical research evidence that looks at single diseases and considers randomized controlled trials (RCTs) to be the gold standard in determining whether a given medicine or device will benefit a specific patient group as compared to no treatment (i.e. placebo) or the current standard of care. However, in the field of public health, where the aim is to improve the health of entire populations, a more complex arsenal of research study designs are needed that better address the complexity and contextual nuances involved, as well as ensuring that research evidence is co-produced with knowledge users who are able to implement changes that in practice will lead to improved health outcomes. Even for diseases where there is a known prevention or cure, people are still dying from these conditions because we lack knowledge on how to make these treatments work in practice in a variety of contexts. The purpose of this article series is therefore to describe how to produce evidence for improving the health of populations and how to ensure that this evidence is then used to make better informed decisions for health. The first article in this series focuses on the different kinds of study designs and approaches that can be used, beyond the traditional focus on RCTs, for producing evidence that can help to improve population health and reduce health inequities.

## Review

**Fig. 1:**
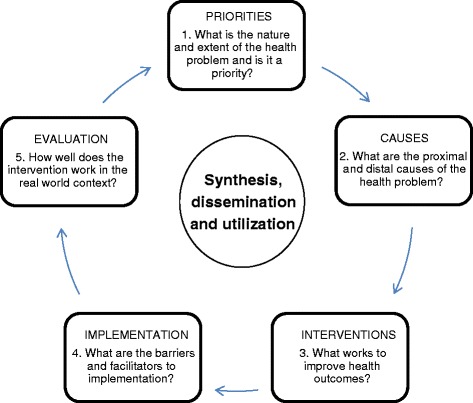
**The research cycle: priorities, causes, interventions, implementation, evaluation [** 1 **]**

It may appear self-evident, but the type of research studies needed to build up the evidence base on how to improve population health and reduce inequities depends on the research questions being asked. For instance, if you want to know the most pressing health priorities in a given population, then you cannot use an RCT to answer this research question. Rather, you might use a cross-sectional survey, a longitudinal panel or a qualitative interview study with key informants. Therefore, different types of research studies are needed to answer different research questions at different stages in the research cycle (Fig. 1). Increasingly, research for health is becoming more multidisciplinary and intersectoral in nature to reflect the growing appreciation that improving health requires intervention at multiple levels, including action on the social determinants on health [2]. The research cycle presented here is therefore an iterative process that involves co-production of knowledge between researchers and decision-makers and provides supporting evidence for the series of actions that are required to improve the health of individuals and populations.

### Defining health priorities

The first step in improving the health of individuals and populations is better understanding what the main health problems are, and of these, which are the most urgent priorities and why. According to the PRECEDE-PROCEED model [3], quantitative data can be used to create a health portrait of the frequency and severity of context-specific health problems (i.e. the ‘objective health needs’), but qualitative data is also needed to explore the perceptions of whether these health problems are considered by the local population to be a priority and why (i.e. the ‘subjective health needs’). For instance, the US National Health and Nutrition Examination Survey [4], and similar surveys in other countries, ask about disease prevalence and may also include direct assessments of the health condition (e.g. identifying diabetes by doing blood sugar tests). However, in addition to knowing about how many people have the health problem and how many new cases develop each year, it is also important to know the severity of the health problem. This, in turn, has important implications for the health system, especially in relation to non-communicable diseases, including mental health conditions, addictions, gender-based violence, child maltreatment and other chronic problems that can cause prolonged suffering, greatly impacting quality of life and increasing the need for care over long periods of time, even if there may not be a significant impact on mortality. To better understand in what ways health problems actually affect people, it is necessary to ask people directly. Therefore, qualitative research can be used to tease out how a health problem impacts people’s lives and what kind of support would be most helpful.

When prioritizing which health problems should be the focus of further research (i.e. moving to step 2 in the research cycle) it is not sufficient to simply make a ranking of the health conditions which result in the largest number of deaths, disability-adjusted life years or which cost the most money. According to Green and Kreuter’s “Precede-Procede” model for health planning, in addition to the ‘objective needs assessment’ based on surveillance data and descriptive surveys, there should also be a ‘subjective needs assessment’ that considers the viewpoint of the local population [5]. People want to be involved [6], and their voices should be heard to ensure a fair process [7] since these decisions will ultimately affect them. Qualitative research is more participatory and inclusive by using purposive sampling to obtain a wide range of perspectives, including those in the minority who may be more marginalized. Thus, it is an important way of involving various populations or target groups in providing their own views and empowering them in determining their own health priorities and identifying their preferred solutions [8].

### Understanding the causes of the health problem

Once the major health priorities are identified, the next step is to better understand the causes of the health problems as a basis for identifying effective interventions. Epidemiological studies, such as case-control and cohort studies, can demonstrate whether there is an association between an exposure (such as smoking) and an outcome (such as lung cancer) [9, 10].

**Fig. 2:**
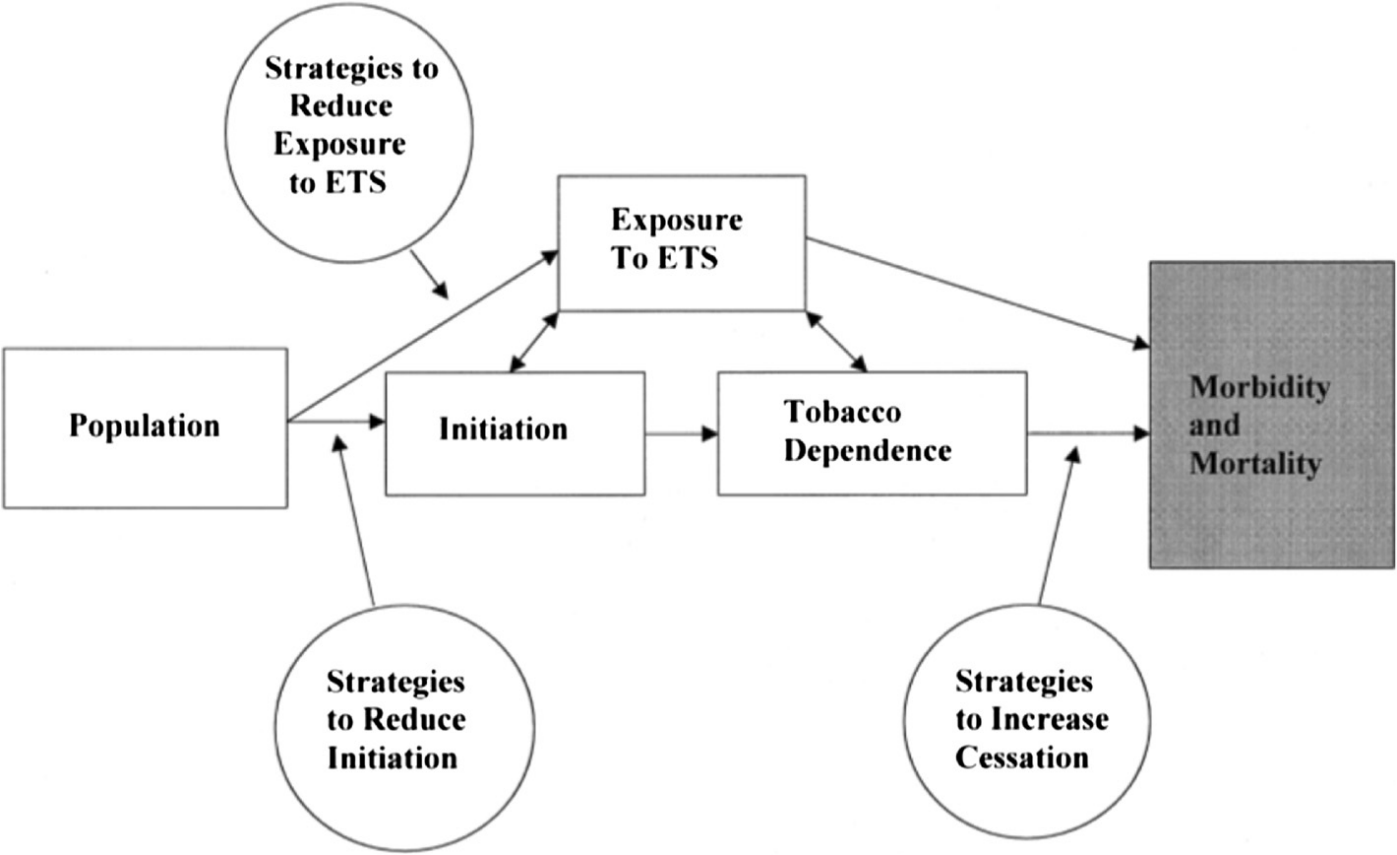
**Logic model to reduce tobacco use and exposure to environmental tobacco smoke.** Adapted from [13]

However, the causes of health problems are often complex and involve a number of proximal risk factors as well as upstream determinants of health. For instance, smoking (i.e. a risk factor) causes lung cancer, but what causes people to start smoking in the first place, and to continue smoking for decades? Indeed, there is a whole literature on the various factors such as peer pressure, marketing and social norms which influence young people to start smoking [11]. Even non-smokers are at risk of disease and therefore need to be protected from environmental tobacco smoke [12]. A ‘logic model’ can explain the complex relationship between the various causal factors and the health problem as a starting point for developing interventions to target these causes. For instance, the Task Force on Community Preventive Services developed a logic model for interventions to prevent the initiation of smoking, to promote smoking cessation and to reduce exposure to environmental tobacco smoke as a means of reducing disease incidence and mortality (Fig. 2) [13].

The next step in the research cycle is determining what works to improve health. This involves developing new interventions or identifying existing interventions that act on the causes of poor health, and then conducting further research to assess which of these interventions actually makes a difference in improving health outcomes.

### Developing interventions to improve health outcomes

In developing and testing interventions, we want to know whether the intervention works, how well it works, whether there are any unwanted negative consequences, whether the benefits of the intervention outweigh the harms, and how much this will cost per incremental improvement in health. While RCTs have long been the gold standard for determining the efficacy of an intervention, these studies can nonetheless have certain methodological challenges that can affect the internal and external validity, and hence the usefulness of the results [14]. This led to the development of the CONSORT reporting standards to at least be able to better judge these shortcomings and determine the utility of the data for decision-making [15]. However, even beyond issues relating to validity of results, RCTs can be extremely expensive, less amenable to studying more complex interventions at the health system or population level, and are also subject to important ethical considerations [16, 17], which are beyond the scope of this article. Therefore, alternative study designs are important and increasingly being used, and include pre-post studies where the population serves as its own control group and stepped-wedge designs with sequential roll-out of interventions over time [18], both of which offer certain advantages, but like all research studies, also have their limitations [19].

### Implementing and evaluating research in a real-world context

The final steps in the research cycle are traditionally the implementation and evaluation of the intervention in ‘real world’ settings rather than controlled research settings. Nevertheless, in population health research, where interventions are often complex and where it can be difficult to find ‘controlled settings’, the boundaries between the development of interventions, and their implementation and evaluation, can be blurred. Ultimately, what we really want to know is whether an intervention has improved the health of the population and has reduced health inequities.

Commonly used ways of assessing whether there has been a positive change in population health are quasi-experimental studies such as pre-post studies, natural experiments and stepped-wedge designs. If an intervention has been shown to produce a positive impact on health, policymakers would also want to know how much the actual implementation of the intervention will cost, and what the incremental cost per additional health benefit produced would be. Economic evaluations can attempt to provide this information, though often rely upon modelling based on a variety of assumptions which may or may not reflect the reality in a given context. Moreover, demonstrating that an intervention is inexpensive and able to produce a health benefit in a controlled research setting is very different from ensuring that the health benefit can be realized within a given budget when the intervention is implemented on a larger scale in a ‘real world’ setting.

The tail end of the research cycle which deals with implementation and evaluation is a grey zone where research blends into practice. In research, the purpose is to generate new knowledge and to develop and test hypotheses. This entails using the various research study designs described above and it also requires ethical approval to protect research participants (regardless of the study design chosen, since all studies pose certain ethical challenges that should not be overlooked) [20]. In contrast, the purpose of implementation and evaluation is to improve the effectiveness and efficiency of programs and policies by modifying, adapting and adjusting these in accordance with lessons learnt from actually using them (and studying how they work) in practice.

According to WHO, most research to date has focused on the development of new interventions rather than optimizing the delivery of existing interventions. There is therefore a call for more research that “*focuses on studying how research outcomes can be translated into practice*” [21]. Of course, the type of research that succeeds in being funded reflects the priorities established by funding agencies, which tend to be overly concerned with developing new technologies and securing intellectual property agreements rather than optimizing delivery and utilization in local contexts. According to Leroy et al. [22], “*ninety-seven percent of grants were for developing new technologies, which could reduce child mortality by 22%. This reduction is one third of what could be achieved if existing technologies were fully utilized*”. Indeed, many evidence-based innovations fail to generate the expected health impact when transferred to communities in the global South, largely because their implementation is untested, unsuitable or incomplete [23]. If the goal of research is improving population health and saving lives, then funding agencies need to rethink whether they are investing in the right places.

In an attempt to maximize the health impact of research by optimizing delivery and utilization of existing technologies, WHO developed an implementation research platform to better understand the challenges of generalizing research findings in the real world and contextualizing interventions for implementation in specific settings [24]. Similarly, evaluation research is intended to assist decision-makers in making better informed choices about whether or not to continue, modify or discontinue a certain policy or program. Not only is this important for performance management by demonstrating accountability, transparency and the judicious use of public funds [25], but ultimately, evaluation research is important to ensure that the interventions implemented are indeed improving the health and well-being of individuals and populations. Thus, greater investment and infrastructure is required to ensure that such research takes place, since far too many programs and policies are put into place without much attention to the underlying evidence base, and are then left in place for years or even decades, with little or no continuous quality improvement to ensure that they are producing the outcomes initially intended.

## Conclusions

There are many different types of research studies that can help to answer a wide variety of research questions. However, in practice, there are certain types of studies that generally prevail, whereas other types are few and far between. For instance, until recently, there was relatively little work in the area of implementation and evaluation research as most research was focused on earlier stages of the research cycle. Indeed, researchers would develop research protocols, apply for funding, conduct their research studies to measure disease or understand causes or test simple disease-specific interventions, prepare manuscripts for publication in high-impact peer-reviewed journals often concluding that “*more research is needed*”, and then start the process all over again – essentially bypassing the implementation and evaluation stages. Therefore, organizations which support research must acknowledge the importance of implementation and evaluation research and provide the necessary resources to develop research capacity and support submitted proposals to strengthen the knowledge base in these fields of research.

It is also increasingly being recognized that research evidence will have very little effect if it does not reach the local knowledge users who are in a position to apply this information to motivate change. Ideally, according to Parry et al. [26], these knowledge users and decision-makers should be engaged in the research process from the very outset, to help inform the key knowledge gaps that need to be addressed and to then ‘translate’ the evidence into policy and practice. This increased emphasis on ‘integrated knowledge translation’, also known as co-production of research evidence, certainly requires more time and effort to build up the required interdisciplinary and intersectoral partnerships, but it also increases the chances that the research findings will be applied and used in practice and will yield tangible results in the long run. Integrated knowledge translation implies that researchers must play an important role in helping knowledge users frame health priorities in a way which can be addressed by the different kinds of research study designs available, often requiring mixed methods approaches to tease out complex issues.

Even if researchers are progressively being encouraged to think about how the research findings can be applied in practice and can now apply for a growing number of knowledge dissemination grants, there nonetheless remain perverse incentive systems in the way that research is funded which lead to some types of research being prioritized over others; not because it is more important nor because it will lead to more significant health gains, but due to the way in which the research enterprise is structured. In this regard, researchers, especially in academic settings, tend to focus on ‘publications’, ‘professorships’ and ‘patents’, rather than ‘policy’, ‘practice’ and ‘people’ [27]. Indeed, the evidence base is hugely biased towards basic science and clinical research (e.g. the effect on blood pressure from using anti-hypertensive medications) rather than population research (e.g. the impact of grassroots community development and social norm modification on the incidence of family violence and child maltreatment). Pratt and Loff [28] further argue that research legislation and policies used in high-income countries have increasingly led these countries to invest in health research aimed at boosting national economic competitiveness rather than reducing health inequities and that the ‘gadget health’ approach “*diverts funding away from research that is needed to implement existing interventions and to strengthen health systems, i.e. health policy and systems research*”.

To ensure that we do not lose sight of the true goals of health research, it is important to look at the big picture and not be blinded by academic or commercial interests, such as the ‘publish or perish’ imperative or the hype surrounding new technologies [29]. There are no ‘magic bullets’ or easy cures for the world’s health problems, which are largely a reflection of underlying economic, social, cultural and political problems. Further, there is little point in producing all of this research evidence if it is not used to make better-informed decisions and policies to improve health. Incentive systems, such as greater availability of funding mechanisms and research awards tailored to this area, and which recognize the importance of applying research in practice, are therefore required. Beyond the amount of publications produced, what if researchers were instead judged based on their efforts to inform the development, implementation and evaluation of policies and programs that prevent human suffering, save lives and reduce inequities? Perhaps then we really would see the benefits of research in practice.
